# Long Non-Coding RNAs, the Dark Matter: An Emerging Regulatory Component in Plants

**DOI:** 10.3390/ijms22010086

**Published:** 2020-12-23

**Authors:** Muhammad Waseem, Yuanlong Liu, Rui Xia

**Affiliations:** 1 State Key Laboratory for Conservation and Utilization of Subtropical Agro-Bioresources, South China Agricultural University, Guangzhou 510640, China; m.waseem.botanist@gmail.com (M.W.); liuyuanlong@scau.edu.cn (Y.L.); 2 Guangdong Laboratory for Lingnan Modern Agriculture, South China Agricultural University, Guangzhou 510640, China; 3 Key Laboratory of Biology and Germplasm Enhancement of Horticultural Crops in South China, Ministry of Agriculture and Rural Affairs, South China Agricultural University, Guangzhou 510640, China

**Keywords:** lncRNAs, dark matter, regulator, database, epigenetics, tolerance

## Abstract

Long non-coding RNAs (lncRNAs) are pervasive transcripts of longer than 200 nucleotides and indiscernible coding potential. lncRNAs are implicated as key regulatory molecules in various fundamental biological processes at transcriptional, post-transcriptional, and epigenetic levels. Advances in computational and experimental approaches have identified numerous lncRNAs in plants. lncRNAs have been found to act as prime mediators in plant growth, development, and tolerance to stresses. This review summarizes the current research status of lncRNAs in planta, their classification based on genomic context, their mechanism of action, and specific bioinformatics tools and resources for their identification and characterization. Our overarching goal is to summarize recent progress on understanding the regulatory role of lncRNAs in plant developmental processes such as flowering time, reproductive growth, and abiotic stresses. We also review the role of lncRNA in nutrient stress and the ability to improve biotic stress tolerance in plants. Given the pivotal role of lncRNAs in various biological processes, their functional characterization in agriculturally essential crop plants is crucial for bridging the gap between phenotype and genotype.

## 1. Introduction

Approximately 90% of the genome is pervasively transcribed into RNA. However, only 1–2% of the transcribed genome encodes for putative protein-coding RNAs, whereas the remaining 98–99% RNAs remain uncharacterized or do not code for any functional protein and are regarded as “dark matter,” transcriptional “noise,” “junk DNA,” or an “experimental artifact” [1,2,3]. Such RNAs are considered to be non-coding RNAs (ncRNAs) [1]. These ncRNAs are of three types: regulatory ncRNAs, circular ncRNAs, and structural ncRNAs. The regulatory ncRNAs are grouped into two sub-groups based on their length: (1) small RNAs (sRNAs), which are 20–31 bp in length, including small interfering RNAs (siRNAs: 19–25 bp), piwi-interacting RNAs (piRNAs: 26–31), and microRNAs (miRNAs: 20–22 bp); and (2) long regulatory RNAs (long non-coding RNAs (lncRNAs) > 200 bp) [4]. Structural or housekeeping ncRNAs consist of transfer RNAs (tRNAs), ribosomal RNAs (rRNAs), small nucleolar RNAs (snoRNAs), and small nuclear RNAs (snRNAs) [5]. CircRNAs (circRNAs) have been recently discovered as a new group of ncRNAs, generally generated by alternative back splicing of pre-mRNA [6].

lncRNAs constitute approximately 80% of total ncRNAs and are thought to play a significant role or act as a critical component in various biological processes [7]. lncRNAs are > 200 bp in length and do not have discernible coding potential. However, lncRNAs share similarities with mRNAs, such as transcription by RNA polymerase II (Pol II), a cap structure at the 5′-end and 3′-poly (A) tail, and splicing [8]. Pol II transcribes most lncRNAs, whereas plant-specific RNA polymerases Pol IV/V can produce lncRNAs [9]. The lncRNAs can be regarded as gene regulatory factors and contribute to three modes of gene expression regulation—transcriptional, post-transcriptional, and epigenetic [4,10]. A significant proportion of lncRNAs (42%) have been found to be involved in transcriptional regulation through transcriptional interference. However, post-transcriptional regulations occur in the cytoplasm, whereas transcriptional and epigenetic modifications occur in the nucleus [11].

Plant lncRNAs are involved in diverse biological processes, including photomorphogenesis, flowering, phosphate homeostasis, stress tolerance, and fertility [9,12]. They also play a vital role as a molecular sink for the target mimics of miRNAs to process into shorter ncRNAs [13], in protein subcellular re-localization [14], as repressors of histone acetylation, in direct epigenetic silencing mediated by specific chromatin domains [15], and in regulating protein post-transcriptional regulation [13,16]. This review briefly delineates the general characteristics of lncRNAs, including classification, progress in annotation, and key underlying mechanisms in plant development and stress tolerance. We aim to elucidate the lncRNA-mediated regulation of important plant developmental processes such as vernalization, fertility, lateral root development, photomorphogenesis, nodulation, and fiber development, and provide guidance for developing optimal plant breeding strategies in future crop breeding programs.

### 1.1. Discovery of lncRNAs

**Figure 1:**
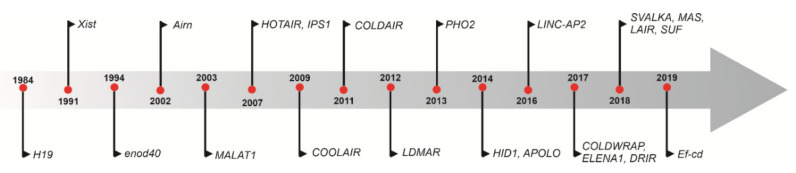
Timeline of the discovery of some of the important regulatory long non-coding RNAs (lncRNAs) in RNA biology from 1984 to 2019. The first lncRNA was the imprinted *H19* gene, followed by the discovery of the XIST lncRNA gene during the pre-genomic era. In plants, lncRNA endo40 was first discovered to be involved in nodulation. Later, *COOLAIR, COLDAIR*, *LDMR*, *PHO2*, *APOLO*, *LINC*-*AP2*, MAS, and other lncRNA discoveries provided insights into their distinct biological roles in different processes such as vernalization, male sterility, sex-differentiation photomorphogenesis, and phosphate (Pi) uptake.

During the pre-genomics era, the lncRNA *H19*—a highly conserved transcript with a length of 2.5 kb—was first discovered in mice in 1984, and was noted to be highly abundant in embryonic cell lineage (Figure 1) [17]. H19 is located upstream of insulin-like growth factor 2 (*Igf2*) gene on chromosome 11 in humans, and on chromosome 7 in mice; they are coregulated and share a common imprinting mechanism [18]. Since this discovery, with the advent of high throughput sequencing, several regulatory lncRNAs such as *Airn, HOTAIR, MALAT1,* and X-inactive-specific transcript (*Xist*) have been discovered and further characterized. The first plant lncRNA, EARLY NODULIN 40 (*ENOD40)*, was isolated from nodule primordia in *Medicago* plants and found to be involved in symbiotic nodule organogenesis [19]. *ENOD40* can induce cytoplasmic localization of nuclear RNA binding protein *MtRBP1* in *Medicago truncatula* during nodule formation [20]. Subsequently, several researchers identified and characterized lncRNAs as a regulator of flowering time [21], anther development and male fertility [22,23], innate immunity [24], nutrient deficiency, and toxicity [25] (Figure 1).

### 1.2. Biogenesis of lncRNAs

**Figure 2:**
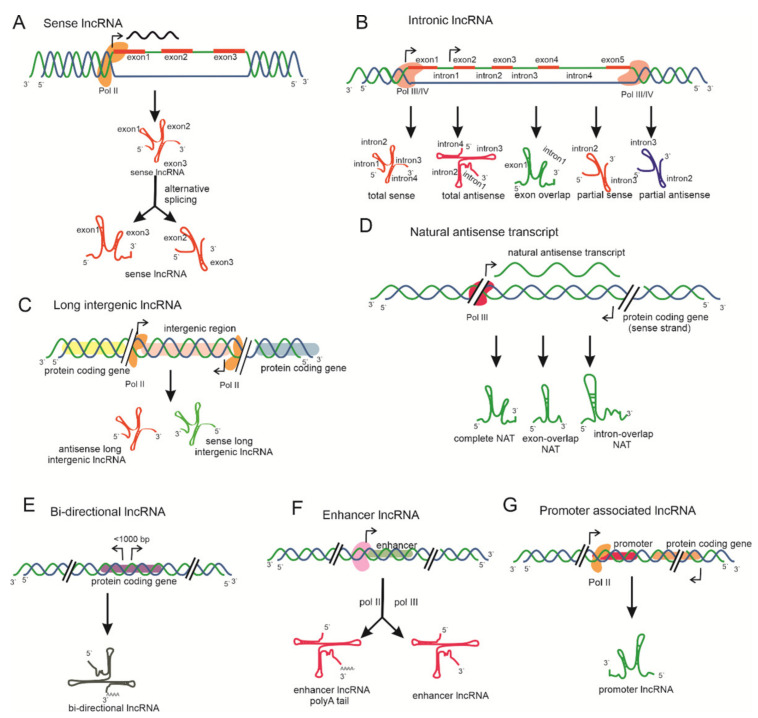
Biogenesis of lncRNAs and their classification based on genomic context. (**A**) Sense lncRNAs transcribed from protein-coding transcripts in the sense and antisense directions by alternative splicing. (**B**) Intronic lncRNAs synthesized from the intronic region, sometimes including exonic sequences. These lncRNAs are also transcribed in both the sense and antisense directions of their corresponding transcripts. (**C**) Those lncRNAs transcribed from intergenic regions of protein-coding transcripts are either in the sense or antisense direction between two protein-coding genes and are designated as intergenic lncRNAs. (**D**) Natural antisense lncRNAs arise antisense to the corresponding protein-coding gene. (**E**) Bi-directional lncRNAs, synthesized from the promoter of the protein coding-gene but in the opposite direction. (**F**) Enhancer lncRNAs arise from the enhancer region of the protein-coding gene, with or without the 3′-poly(A) tail modification. (**G**) LncRNAs transcribed from the promoter region of protein coding-genes, controlling the transcriptional regulation of related genes.

lncRNAs belong to a heterogeneous subclass of > 200-nt ncRNA transcripts lacking coding potential and appear as an emerging field of research that has been accelerated and expanded in the last decade. lncRNAs act as riboregulators and are localized in both the cytoplasm and nucleus and are transcribed by RNA polymerase II or III or IV/V [26,27,28]. RNA Pol II transcribes lncRNAs with a 5′-cap and poly-adenylation at the 3′ tail from various genome regions, including enhancer or intron splicing regions [29]. RNA pol IV and V generate lncRNAs through RNA-dependent DNA methylation (RdDM), which serve as precursors for small interfering RNAs (siRNAs) [30]. lncRNAs, with their possible biogenesis, are illustrated in Figure 2.

### 1.3. Status of lncRNAs in Plants

To date, a plethora of putative lncRNAs have been predicted in various plant species, such as Arabidopsis thaliana, Solanum lycopersicum, Zea mays, Medicago, Oryza sativa, Solanum tuberosum, Triticum aestivum, Populus trichocarpa, Setaria italica, and Brassica rapa (Table 1). The number of identified lncRNAs varies considerably, ranging from 6480 in A. thaliana to 58,218 in S. tuberosum [11]. All of these putative lncRNAs play various roles in response to different biotic stresses such as tomato yellow leaf curl virus (TYLCV), infection by Sclerotinia sclerotiorum, Fusarium oxysporum, and Puccinia striiformis, as well as to abiotic or environmental stresses like drought, low and high temperatures, osmotic or hypoxic stress, and nitrogen or phosphate deficiency [11]. In Camelina (Camelina sativa L.), through drought-stress specific transcriptome analysis, 5390 putative lncRNAs were identified, including 1347 intergenic, 69 antisense, 670 sense, and 2681 intronic lncRNAs [31]. Mining more than 200 transcriptome datasets for Arabidopsis [32] revealed that 70% of lncRNAs (~40,000 candidate lncRNAs; >30,000 natural antisense transcripts (NATs); >6000 long intergenic ncRNAs (lincRNAs)) were transcribed from coding loci to be associated with antisense transcripts [33,34]. Similarly, large-scale transcriptome and genome resequencing between rice and its closely related wild relatives (858 accessions) revealed 3363 lncRNAs in developing panicles. Among these, 329 lncRNAs were downregulated [35]. Genome-scale lncRNAs annotation from 749 RNA sequencing experiments on Z. mays encompassing 17 different developmental stages uncovered 18,165 lncRNAs in Z. mays, of which 37.83% lncRNAs were conserved between maize and teosinte [36] These significant differences in expression strongly suggest that lncRNAs diversified during the domestication of crop plants. A total of 1299, 1885, and 1910 lncRNA candidates in B. rapa, B. napus, and B. oleracea were identified, respectively [37]. Mining the rice whole transcriptome RNA sequencing libraries, 2588 lncRNAs under nitrogen starvation [38] and 2224 putative lncRNAs encoding loci associated with the reproductive process were verified [39]. Moreover, a total of 682 lncRNAs in cassava [40], 7245 lncRNAs in Z. mays L. [41], and 2542 lncRNAs in P. trichocarpa [42] were identified. These studies of lncRNAs in plants laid the foundation for the functional characterization of lncRNAs and their potential role in growth, development, and tolerance to various environmental stresses [11,43].

In the past two decades, significant progress in plant lncRNA prediction and functional characterization has been achieved [44]. In planta, some important plant lncRNAs such as phosphate-limitation inducible gene 1 (*OsPI1)*, trehalose phosphate synthase 11 (*TPS11*), *ENOD40,* induced by phosphate starvation 1 (*IPS1*), cooled assisted intronic non-coding RNA (*COLDAIR)*, cold induced long antisense intragenic RNA *(COOLAIR)*, and long day (LD)-specific male-fertility-associated RNA (*LDMAR*) have provided insights into their distinct biological roles in various fundamental processes including vernalization, male sterility, nodule formation, photomorphogenesis, and phosphate (Pi) uptake [19,21,45,46,47]. For instance, *ENOD40* was the first plant lncRNA predicted to act as a riboregulator in *M. truncatula, Oryza sativa, Glycine max,* and during root nodule organogenesis [20]. *COOLAIR* and *COLDAIR*, antisense intronic lncRNA transcripts of flowering locus C (*FLC*), function as epigenetic repressors of *FLC* transcription during vernalization in *Arabidopsis* [46]. Phosphate 2 (*PHO2*) plays a critical role in phosphate signaling and is one of the targets of miR399 [48]. Phosphate-starvation-inducible lncRNAs *At4* and *AtIPS1* act as target mimics of miR399 and sequester miR399 to reduce miR399-mediated cleavage of *PHO2* [45,48]. These findings suggest that lncRNAs may act as essential regulatory molecules in moderating plant responses to environmental changes [21].

There have been many efforts in the development of computational tools and high-throughput sequencing technologies, such as RNA capture sequencing (RNA CaptureSeq), whole-genome tiling arrays, the lncRNA microarray technique, serial expression of gene expression (SAGE), the expressed sequence tag (EST) method, and RNA-sequencing (RNA-seq). More than half a million lncRNA transcripts have been predicted in silico to understand their biological mechanisms [49]. Accumulating evidence suggests that lncRNAs are emerging regulators of diverse biological processes.

## 2. Annotation of lncRNAs

Next-generation sequencing (NGS) technology and computational approaches enable us to generate transcript fragments and align them with a reference genome to identify novel RNAs. Likewise, mining RNA-seq data can help readily identify lncRNAs. However, it is challenging to differentiate the coding RNA from non-coding RNAs in thousands of assembled transcripts [50].

### 2.1. Computational Tools and Resources for lncRNA Identification

In plants, some studies have used both experimental and computational techniques to identify and characterize lncRNAs. Some important computational techniques to distinguish ncRNAs from protein-coding transcripts (Table 2), including coding or non-coding (CONC) for analyzing large ncRNA datasets; and coding potential calculator (CPC: score <0) for known protein-coding transcripts [51]. PhyloCSF distinguishes coding transcripts from non-coding ones using codon substitution frequency (CSF) scores. The Coding-Potential Assessment Tool (CPAT) is based on a logistic regression model, whereas Coding-Non-Coding Index (CNCI) and CPC are based on the support vector machine (SVM) [52,53,54]. To identify lncRNAs from transcriptome sequencing data, two standalone tools based on deep neural networks (DeepLNC) and the SVM algorithm (iSeeRNA) have also been developed [55,56].

Moreover, bioinformatics researchers have developed several tools to predict the mechanism of lncRNA regulation (Table 2). lncRNA–mRNA/–DNA interactions based on nucleic acid thermodynamics can be predicted using the lncRNATargets platform [57]. lncRNA–miRNA interactions and regulatory RNA motifs/functional sites, as well as common transcription factors for lncRNAs can be identified using spongeScan, RegRNA, and TF2LncRNA, respectively [58,59,60].

### 2.2. Databases for lncRNAs

Over the past decade, the number of discovered lncRNAs has significantly expanded. Thus, there is a need to organize the identified lncRNAs with their biological functions in a user-friendly environment. Several such databases have been archived for identified lncRNAs (Table 1). PLncDB integrates large-scale multi-omic datasets of lncRNAs such as RNA-seq, tilling array, siRNAs, and epigenetics. It contains 16,227 lncRNAs identified from the *A. thaliana* genome only [32]. PNRD is a collection of nearly all kinds of ncRNAs, including lncRNAs in rice, *Arabidopsis*, maize, and poplar [65]. lncRNAdb is a database for a few plant species, such as *A. thaliana*, *O. sativa*, *Vitis vinifera*, *M. truncatula*, *B. rapa G. max*, *S. lycopersicum*, and *Populus tremula*. lncRNAdb provides comprehensive annotations for eukaryotic lncRNAs, including evolutionary conservation, gene expression data, genomic context, structural information, functional evidence, subcellular localization, and the transcript sequence and their experimentally verified biological functions [64]. NONCODE (V6) is an interactive database that presents a complete collection and annotation database of ncRNAs in 39 species, including 23 plants, such as *Arabidopsis* with 3796 lncRNAs, *O. sativa* with 1123, and *Z. mays* 4279 [62]. It provides basic information about lncRNA expression profiles and predicted functions. GREENC provides a comprehensive annotation of more than 120,000 plant-specific lncRNAs from 37 plant species. This database provides an inbuilt pipeline for lncRNA prediction and evaluates coding potential and folding energies of lncRNAs [63]. PLNlncRbase is a repository of 1187 experimentally verified lncRNAs in 43 plant species collected from approximately 200 publications [67]. CANTATAdb includes several computationally identified lncRNAs from 10 model plant species [66]. PlncRNADB contains 5000 lncRNAs from *Arabidopsis*, *Z. mays,* and *Populus*, serving as a reference database for lncRNA prediction and evaluating the coding potential of protein transcripts. It also contains several computational pipelines for lncRNAs. It is worth mentioning that PlncRNADB displays the relationships between lncRNAs and various RNA-binding proteins (RBPs), which can be visualized as lncRNA–protein networks [68]. The EVLncRNAs database contains annotatedand experimentally validated lncRNAs—1543 lncRNAs from 77 species, including 428 plant lncRNAs from 44 plant species developed by Zhou et al. [69].

## 3. Classification of lncRNAs

lncRNA classification is a challenging task that is under active development and is so far based on several distinct features—genomic location and context, mechanism of function, targeting mechanism, and effect exerted on DNA sequences. However, the classification of lncRNAs is immature because their state of annotations is at its beginning, and classifications based on transcript properties and their length, the orientation of their regulatory elements in relation to known genomic annotations and functions have been proposed [70]. We summarize lncRNA classification based on genomic context, including the orientation relative to nearby protein-coding genes and function modes.

### 3.1. Based on Genome Context

Based on the genomic context, lncRNAs are transcribed from various genomic loci, including sense and antisense orientations of coding genes, intergenic regions, promoter regions, and enhancers [12]. These lncRNAs are categorized as natural intronic RNAs (incRNAs), antisense transcripts (lncNATs), bidirectional lncRNAs (BI-lncRNAs), overlapping lncRNAs (OT-lncRNAs), and long intergenic ncRNAs (lincRNAs) (Figure 2) [71]. LncRNAs transcribed from protein-coding regions of a gene and of which the promoter region may be retained from a few or all of the protein-coding exons by alternative splicing are designated as sense lncRNAs (Figure 2A). Those originating exclusively from the intronic region, sometimes including the exonic region, of protein-coding genes are known as intronic lncRNAs (Figure 2B) [72]. Sense lncRNAs lack protein-coding potential; their expression levels are lower than their corresponding mRNAs [73]. *COLDAIR* is a well-studied example of sense and intronic lncRNAs in plants. Intergenic lncRNA is transcribed from intergenic regions of protein-coding genes either in a sense or an antisense orientation (Figure 2C). Such lncRNAs act as a precursor for other ncRNAs. A total of ~2000 intergenic lncRNAs have been identified in various plant species, which are unique, functionally diverse, and are a major focus of lncRNA research. *ENOD40* [74] and *LDMAR* [47] are the two most famous examples of such lncRNAs. Those lncRNAs that originate in the relatively opposite direction to the protein-coding gene are categorized as natural antisense transcripts (NATs) or antisense lncRNAs (Figure 2D) [33].

NATs play a major role in the transcriptional and post-transcriptional regulation of their corresponding protein-coding gene. NATs may act in cis or in trans to regulate gene expression or the array of functions in plants. *COOLAIR* is a cis-acting antisense lncRNA required for regulating flower time through the silencing of *FLC* [75]. *Hidden treasure 1* (*HID1*) is a trans-acting gene involved in photomorphogenesis in *Arabidopsis* seedlings by suppressing phytochrome-interacting factor 3 *(PIF3)*, a key repressor of photo-morphogenesis [76]. However, other types of lncRNAs also exist based on their origin in the genome. These include bidirectional lncRNA, enhancer lncRNA, and promoter-associated lncRNA (Figure 2E–G). Bidirectional lncRNAs are transcribed from transcripts in close proximity (<1000 bp) to the transcription start site of protein-coding genes but proceed in the opposite direction (Figure 2E) [77]. Enhancer lncRNAs are synthesized from the enhancer region of protein coding genes with or without a poly-(A) tail and mediate transcriptional regulation of protein coding genes (Figure 2F) [78]. Lastly, lncRNAs originate from promoter region protein coding genes and control the regulation of corresponding protein coding genes (Figure 2G) [79]. *COLDWRAP* is a 316-nt lncRNA, transcribed 225 nt upstream from the *FLC* gene [80].

### 3.2. Based on the Criteria of Functional Mode

**Figure 3:**
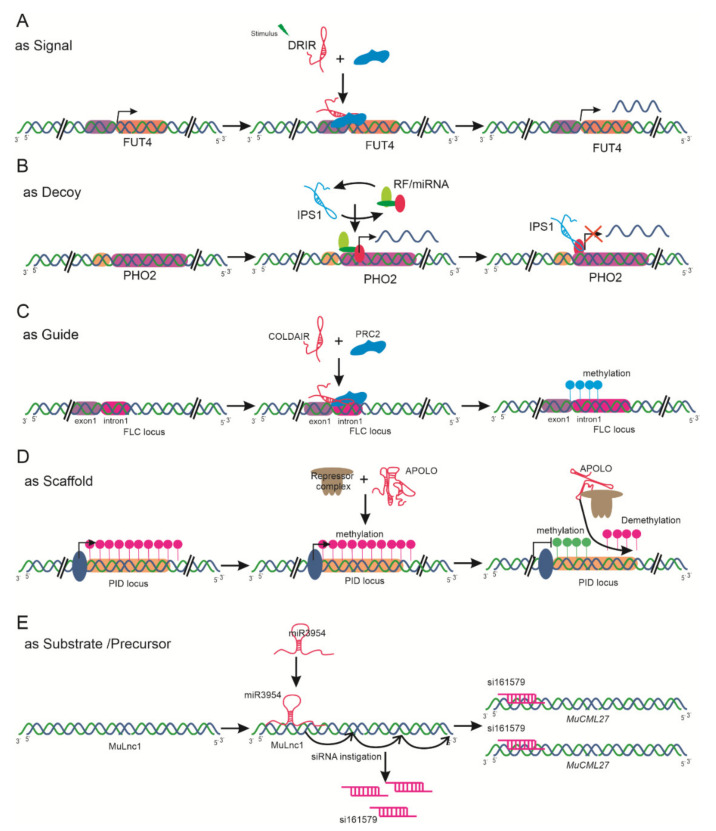
lncRNA classification based on their mechanisms of action—(**A**) as signal molecules to activate the transcription of the protein-coding transcript under a specific stimulus; (**B**) as decoys binding to miRNA or regulatory factors (RFs) and subsequently altering the corresponding function; (**C**) as guides, recruiting the chromatin-modifying complex that acts in cis or in trans of the target protein-coding transcript; (**D**) as scaffolds, recruiting multiple protein complexes and participating in chromatin modification to repress the target gene; (**E**) as substrates for miRNA-mediated phased secondary small interfering RNA (phasiRNA) generation, which functions in targeting several downstream genes.

Increasing evidence suggests that lncRNAs play various pivotal roles during plant life, including roles in chromatin remodeling, molecular imprinting, as transcriptional regulators, in RNA processing, and as ribonucleoprotein regulators. Here, we briefly summarize the classification of lncRNAs based on their function reported to date as signals, decoys, guides, scaffolds, and precursors for small interfering RNAs (siRNAs) (Figure 3).

lncRNAs may act as molecular signals in distinct biological processes and integrate developmental cues due to their specificity and diverse spatiotemporal expression in response to different biological stimuli. For instance, In *Arabidopsis*, *COLDAIR* is associated with the repression of *FLC* by recruiting polycomb repressive complex 2 (PRC2). This complex includes the curly leaf (CLF) subunit, possessing histone methyltransferase activity and catalyzing H3K27me3 deposition at FLC [21]. A drought-responsive lncRNA, drought induced lncRNA (*DRIR*), acts to regulate the activity of the *FUT4* gene encoding fucosyltransferase, resulting in enhanced tolerance to drought (Figure 3A) [81].

lncRNAs may act as decoys, in the form of negative transcription regulators. They function as miRNA target mimics and titrating target proteins or regulators. In maize, degradome analysis revealed that 58 lncRNAs act as decoys. In *Arabidopsis*, lncRNAs—including *IPS1*, involved in inorganic P (Pi) homeostasis with *PHO2* [45], and the alternative splicing competitor (*ASCO*) lncRNA—have been demonstrated to interact with miRNAs [82]. Similarly, lncRNAs are predicted to be target mimics for tomato miRNAs in response to infection with TYLCV [83] (Figure 3B).

Some lncRNAs serve as guides to bind or direct ribonucleoprotein complexes to specific target regions to regulate gene expression. *COLDAIR* directs the PRC2-mediated histone H3Lys27 methylation at *FLC* during vernalization in *Arabidopsis* (Figure 3C) [84]. Likewise, the ELF18-induced long-noncoding RNA 1 (*ELENA1*) lncRNA induced by infection of *Pseudomonas syringae* acts in cis/trans, imparting plant immunity through pathogenesis-related gene 1 (*PR1*) [24]. Several lncRNAs may serve in the scaffolding mechanism as a central podium for the assembly of various molecular components. *APOLO*, a polymerase II/V transcribed lncRNA, regulates chromatin loop formation (Figure 3D) [26].

lncRNAs may act as precursors or substrates for siRNAs, negatively regulating the target gene (Figure 3E) [85]. Among these, trans-acting small interfering RNAs (tasiRNAs) or phased secondary small interfering RNAs (phasiRNAs) are two endogenous classes of siRNAs [85,86]. Recently, various studies on lncRNA-derived siRNA havce been reported; among these, the miR390-*TAS3*-ARF pathway [87], the miR3954-lncRNA-phasiRNA-NAC pathway [88], the mul-miR3954-*MuLnc1*-si161579-*MuCML27* [89], and miR2118-*PMS1T*-phasiRNAs [90] are well known in various plant species. For instance, a novel lncRNA, *MuLnc1*, was targeted by miR3954 to produce 21 nt phasiRNAs in mulberries. Among these phasiRNAs, si16157 can cleave calmodulin-like protein gene MulCML27, which plays an important role in response to stress. This suggests that from the complex regulatory network of ul-miR3954-*MuLnc1*-si161579-*MuCML27*, *MuLnc1* may be considered as a candidate target lncRNA for genetic improvement in mulberries [89].

## 4. Role of lncRNAs in Plant Development

Currently, lncRNAs have become a hot topic in the molecular biology community. In plants, lncRNAs belong to a novel class of ncRNAs, and the regulatory mechanism of lncRNAs is scarce. Only 1% of plant-annotated lncRNAs have been characterized, and the emerging shreds of evidence support the fact that lncRNAs play a crucial role as regulators in a wide range of biological processes, including in regulation of mRNA processing and transcription mechanisms, nutrient metabolism, flowering, and male sterility through transcriptional modulation of the target transcript.

### 4.1. lncRNAs Regulate Flowering

Reproductive success of plants tightly linked to the proper timing of floral transition and morphogenesis. Flowering in the plants is controlled by various internal and external cues such as hormones, day length, and temperature. In *Arabidopsis*, vernalization downregulates the expression of the *FLC* gene to promote flowering in spring. Several lncRNA—*COLDAIR, COOLAIR,* and *COLDWRAP*—are involved in the fine-tuning of *FLC* expression. *COLDAIR* and *COOLAIR* are cold-inducible natural intronic lncRNAs of the MADS-box floral repressor transcription factor, *FLC*, which may participate in epigenetic reprogramming and serve as a transcription regulator, respectively [46,84]. *COLDAIR* is a sense lncRNA originating from a cryptic promoter, harboring the first intron of *FLC*. This promoter becomes active upon *FLC* repression. In cold conditions, *COLDAIR* recruits the *PRC2* protein for epigenetic silencing of *FLC* through histone deacetylation of H3K9 and trimethylation of H3K27 modification at the floral repressor gene *FLC* [91].

**Figure 4:**
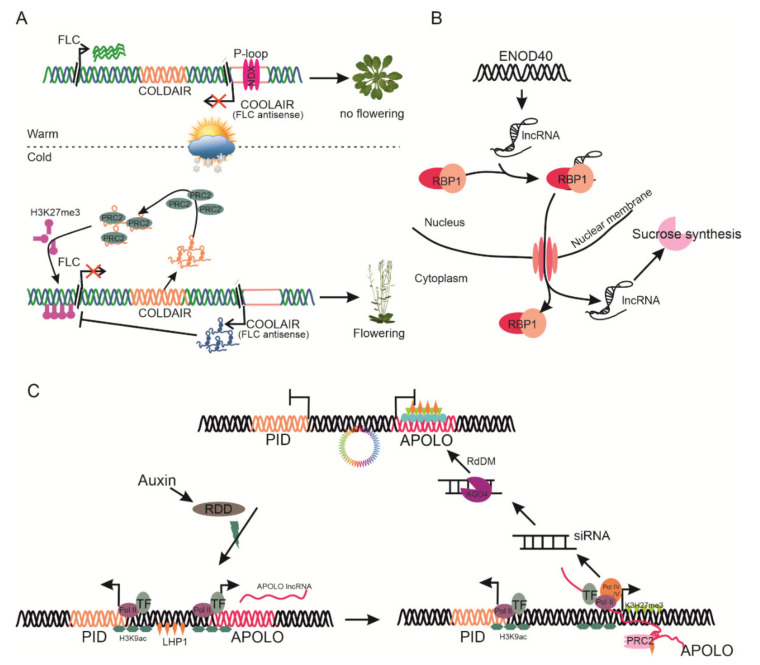
Representative lncRNAs involved in various developmental processes. (**A**) Regulation of gene expression by *COOLAIR* and *COLDAIR* lncRNAs during vernalization. Under high-temperature conditions, the R-loop in the *COOLAIR* promoter is activated by NDX proteins, and the *COOLAIR* transcript binds to the single-stranded DNA, promoting *FLC* locus expression. Upon exposure to cold conditions, *COLDAIR* binds to *PRC2,* whereas *COOLAIR* induces methylation in the *FLC* gene promoter to suppress its transcription. (**B**) The function of *ENOD40* lncRNA in post-transcription of nodulation. (**C**) Regulation of lncRNA *APOLO* produced from the *PID* locus and its subsequent response to auxin polar transportation. In response to auxin, DNA demethylation of *APOLO* lncRNA is activated through chromatin loop activation, and RNA Pol II-transcribed *APOLO* recruits *LHP1*, thus increasing *APOLO* and *PID* expression. In contrast, Pol IV/V activates the PID promoter’s DNA methylation by recruiting AGO4 and PRC1 on the APOLO gene to repress *PID* synthesis.

In contrast, *COOLAIR* is an overlapping antisense-lncRNA which has polyadenylation properties for the *FLC* locus. The proximal 3′ polyadenylation downregulates *FLC* expression through H3K4 demethylation of *FLC* in the cold response, whereas distal polyadenylation activates *FLC* transcript accumulation [92]. *AtNXD* (neoxanthin-deficient 1) is a homeodomain protein with a divergent domain, localized to a heterochromatic region in the *COOLAIR* promoter. *AtNXD* directs and stabilizes R-loop formation in the *COOLAIR* promoter, leading to its downregulation and *FLC* accumulation and facilitating flowering [93]. However, during vernalization, *COLDAIR* directs the suppression of the *FLC* locus, whereas the repression depends on *COOLAIR* [94]. Therefore, during warm days *AtNXD* facilitates the suppression of *COOLAIR* while both *COOLAIR* and *COLDAIR* activate the PRC2 protein as an epigenetic modifier, thus promoting *FLC* expression (Figure 4A). *COLDWRAP* is transcribed from the promoter of *FLC*, recruiting PRC2 to repress *FLC* [80]. MAS is a cis-acting cold-inducible NAT lncRNA of MADS affecting flowering 4 (*MAF4*), localized in nucleus. *MAS* has been found to be present in the *MAF4* locus, which extends from the 5′ transcriptional termination site (TTS) to the first intron at the 3′ end. *MAS* contains a polyadenylation site to bind and recruit WD40-containing repeat 5a (WDR5a) to *MAF4* sites, thus enhancing H3K4me3 and activating *MAF4* expression, fine-tuning the flowering time [95]. CDF5 long non-coding RNA (*FLORE*) is a natural antisense transcript lncRNA of cycling DOF factor 5 (*CDF5*). *CDF5* is involved in circadian clock delay flowering by repressing flowering locus T (*FT*) transcription. *FLORE/CDF5* antiphasic expression reflects mutual inhibitory regulation, which directly affects flowering time regulation. However, *FLORE* directs the activation of FT through repressing *CDF* that promotes flowering [96]. The molecular mechanism underlying this antiphasic regulation needs to be identified in detail.

### 4.2. lncRNAs as a Mediator of Reproductive Development

The floral meristem develops into different floral organs. With advances in functional genomics, especially in RNA-sequencing, researchers have discovered several lncRNAs that control floral and reproductive development. These lncRNAs include early flowering-completely dominant (*Ef-cd*), LDMAR, photoperiod-sensitive genic male sterility T (*PMS1T*), and *LINC-AP2* [47,90,97,98]. *LINC*-*AP2* is an intergenic lincRNA of *APETALA2* (*AP2*) [98]. Plants with *Turnip crinkle virus* (*TCV*) infections showed reduced expression of *AP2* and showed elevated expression of *LINC-AP2*, causing floral structure distortion. *Brassica campestris* male fertility 11 (*BcMF11)* is critical for pollen development and male fertility [23]. LncRNA *AG-incRNA4* is transcribed from the second intron of the agamous (*AG*) gene in *Arabidopsis* and is involved in PRC2 complex component CLF-mediated epigenetic modification of *AG*, thereby contributing to repression of *AG* [99].

In dioecious liverwort, *Marchantia polymorpha,* an MYB-type transcription factor, female gametophyte MYB (*MpFGMYB*), plays a critical role in female sexual differentiation. Loss-of-function *MpFGMYB* results in female-to-male sex conversion. Suppressor of feminization (*SUF*), a cis-acting antisense lncRNA of *MpFGMYB*, is recognized as an important regulator of male sexual differentiation and can suppress its expression only in cis. During sex differentiation, the constitutive expression of *SUF* suppresses *MpFGMYB* expression, encouraging the accumulation of an unknown factor (M), thus subsequently activating Y-chromosome and autosomal genes to develop male gametophyte. On the other hand, a dominant “*FEMINIZER*” on the X chromosome acts through an unknown mechanism to suppress *SUF* expression. Subsequently, it activates the expression of *MpFGMYB* and autosomal and X-chromosomal genes to promote female sexual differentiation. The *MpFGMYB/SUF* locus acts as a bidirectional transcriptional toggle for sex differentiation in liverworts [100]. However, the mechanism by which the *SUF* lncRNA suppresses *MpFGMYB* expression remains uncertain.

A novel lncRNA named *LDMAR* has been suggested to regulate photo-sensitive male sterility in rice [47]. Moreover, a spontaneous point mutation leads to altered RNA secondary structure and subsequently increases DNA methylation in the LDMAR promoter region, which results in programmed cell death in developing anthers. Furthermore, a siRNA named Psi–*LDMAR* is derived from the promoter region of *LDMAR* involved in DNA methylation and represses its expression through RNA-dependent DNA methylation (RdDM) [101]. Genome-scale RNA-seq analysis predicted several lncRNAs, and DNA methylation-regulated lincRNAs from ripening tomatoes have suggested their regulatory role during fruit development and ripening [102,103]. Yu et al. [104] suggest that *lncRNA2155* inactivation delays fruit ripening.

### 4.3. Modulating Other Developmental Processes

lncRNAs play a critical role in protein structure and function modification and cellular localization of target proteins. *ENOD40*, a 700-nucleotide-long lncRNA, participates in nodulation specifically in legumes, and is found in several non-legumes. It encodes two short peptides (*ENDO40*-I: 13 amino acid, *ENDO*-II: 27 amino acid) that bind to sucrose synthase. In this context, this suggested the role of *ENOD40* in sucrose synthase for utilization of sucrose during nodulation [105]. *M. truncatula ENOD40* binds to RNA binding protein 1 (*RBP1*), facilitating its translocation from the nucleus to the cytoplasm. However, *Arabidopsis RBP1* plays a critical role in ABA-mediated stress tolerance and development (Figure 4B) [106].

Hidden treasure 1 *(HID1)* is the first known nuclear-localized lncRNA (236 nucleotides) that negatively regulates photomorphogenesis by binding directly to phytochromeinteracting factor 3 *(PIF3)*, a repressor in photomorphogenesis [76]. auxin regulated promoter loop *(APOLO)* intergenic lncRNA, located about 5 kb upstream of *PINOID* (*PID*), a key polar auxin regulator, is transcribed by RNA Polymerase -II and -V. *APOLO* controls the *PID* gene expression and activates the formation of a chromatin loop surrounding the *PID* promoter. Auxins promote the unwinding of the *APOLO*-*PID* chromatin loop by triggering the demethylation of *APOLO*. RNA Pol II facilitates like heterochromatin protein 1 (*LHP1*) for loop formation. The *APOLO* transcripts produced by RNA Pol II/V recruit *PRC1* and *ARGONAUTE4* (*AGO4*) for loop closing and triggering DNA methylation. Finally, the *APOLO* lncRNA-mediated chromatin loop is reformed and represses *PID* (Figure 4C) [26]. *MIKKI* is a four-intron-containing retrotransposon element that controls root development by regulating the scarecrow like (*SCL*) gene expression. *MIKKI* specifically binds to miR171 to mimic its expression, thus interpreting its root development role [107]. Transcriptome analysis of flower and fruit tissues in *Poncirus trifoliata, Fragaria vesca,* and *Cicer arietinum* demonstrated tissue-specific expression of lncRNAs, indicating their potential role in regulating floral development and fruit ripening [108,109,110].

Conclusively, these findings suggesting the critical functions of lncRNAs in plant reproductive growth through different regulatory mechanisms. The lncRNAs, as mentioned earlier from various plant species, display high tissue-specific expression patterns, suggesting that lncRNAs might play a critical regulatory role in plant development. It is necessary to carry out further studies on the function and mechanism of lncRNAs (Table 3).

## 5. Role of lncRNAs in Biotic and Abiotic Stress

**Figure 5:**
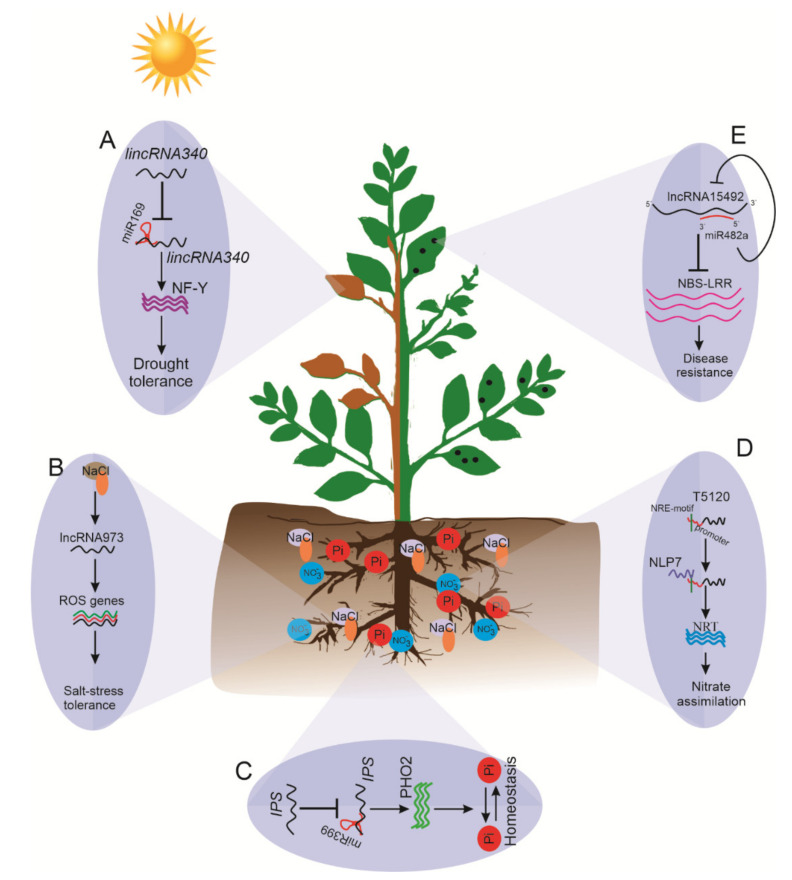
LncRNAs controlling abiotic and biotic stress responses in plants. (**A**) In drought conditions, lncRNA *lincRNA340* is induced to repress miR169, which relieves nuclear factor Y (*NF-Y*) gene expression to improve stress tolerance. (**B**) lncRNA973 acts as a positive regulator of salt-responsive genes in ROS (reactive oxygen species) to enhance salinity tolerance. (**C**) *IPS1* decoys miR399 to regulate the expression of *PHO2* and help maintain phosphate homeostasis in the root. (**D**) lncRNA T5120 functions in NLP7-mediated nitrate regulation in roots. (**E**) The aggressive interaction between Sl-miR482a and *Sl-lncRNA15492* in tomatoes helps to maintain Sl-NBS-LRR homeostasis, thereby affecting the resistance of tomatoes to *P. infestans*.

Globally, changes in the environment due to erratic patterns of seasons, rainfall, temperature, and pathogens affect and pose a serious challenge to global food security. Plants have adopted dynamic responses to such abiotic stresses through various complex molecular networks, including biochemical and physiological cascades of different signal transduction pathways [111]. Subsequently, discovering stress-responsive lncRNAs with their target genes in various plant species has enabled us to understand the molecular mechanism underlying such stress adaptations. Moreover, the large-scale identification of lncRNAs has revealed their participatory roles in stress responses in plants. Such studies have established the pivotal role of ncRNAs in mediating stress tolerance at transcriptional, post-transcriptional modification, and epigenetic levels. As mentioned, a total of 1187 experimental-verified stress-related lncRNAs from 43 plant species have been listed on PLNlncRbase under 17 different biotic and abiotic conditions [67]. In contrast, the regulation mechanism of lncRNAs in response to biotic and abiotic stresses, which allows the plant to survive in a harsh environment, needs to be explored further (Figure 5).

### 5.1. lncRNAs in Drought Stress

Emerging evidence suggests that lncRNAs could directly affect drought responses by regulating the transcription of various stress-responsive genes or by recruiting complex mechanisms such as endogenous target mimics (eTM), chromatin modulation, and antisense transcription-mediated modulation [30]. Recently, genome-wide transcriptome analysis has revealed a variety of stress-responsive lncRNAs in various plant species, including six in *Arabidopsis* [112], *Populus* [42], rice [113], maize [114], foxtail millet [115], cassava [40], and switchgrass [116]. Systematic RNA-seq analysis of *P. trichocarpa* provided the opportunity to explore 2500 lncRNAs of which ~504 lncRNAs were categorized as drought-responsive [42]. To survey the drought-responsive lncRNAs in foxtail millet, deep sequencing served to identify ~584 lncRNAs, of which 17 lincRNAs and 2 NAR lncRNAs were droughts responsive [115]. In cassava, strand-specific RNA-seq data revealed 153 NAT lncRNAs and 318 lncRNAs responding to drought and cold stress, respectively [40]. Considering the regulatory mechanism of lncRNAs, 98 lncRNAs were explored in a comprehensive landscape of the rice genome. Among them, two key drought-responsive NAT lncRNAs—*Os02g0250700–01*, targeting the late embryogenesis abundant protein gene, and *Os02g0180800–01,* targeting the cinnamoyl-CoA reductase gene—were also recovered [113].

Moreover, eTM *lincRNA340* acts as a target mimic for miR169, which controls the expression of the nuclear factor Y *(NF-Y)* gene under drought conditions (Figure 5A) [40]. lncRNAs may regulate the transcriptional expression of various genes. One possible role of lncRNAs as a transcriptional regulator in response to drought has been explored in *Arabidopsis.* A 755-nt-long nuclear-localized lncRNA designed as drought-induced lncRNA (*DRIR*) regulates several drought-responsive genes such as those involved in ABA-signaling (*P5CS1*, *RD29A*/B, and *ABI5*); annexin (*ANNAT7*); and aquaporin genes (*TIP4*, *NIP1*). Moreover, *DRIR* may act to influence the activity of fucosyltransferase 4 (*FUT4*) or transcription factor *NAM/ATAF/CUC 3 (NAC3*), resulting in improved tolerance to salinity and drought stresses (Qin et al., 2017), suggesting that lncRNA *DRIR* may confer drought tolerance by acting as a positive regulator. Conclusively, the emerging drought stress-responsive lncRNAs could act as a target mimic for various stress-responsive miRNAs that regulate different drought-responsive genes and transcription factors. These lncRNAs may act as a critical regulatory hub for various drought-responsive signal transduction pathways at various transcriptional and post-transcriptional levels.

### 5.2. lncRNAs in Salinity Stress

Like other abiotic stresses, salinity stress is one of the major limiting factors for plant growth and productivity. With the aid of “omics” approaches, the role of lncRNAs has been investigated in various plant species. For instance, 3030 lincRNAs and 275 lncNATs were identified from salt-stressed roots of soybean plants [117]. Salt stress also altered the accumulation of lncRNAs in *Arabidopsis* [118] and *Medicago* [119]. Moreover, in grapevine and *C. sinensis*, 1661 lncRNAs and 172 lncRNAs were found to be differentially expressed in roots under salt stress, respectively [120,121]. Similarly, in cotton, 44 lncRNAs were differentially expressed, of which *lncRNA973* was observed to regulate salt stress-responsive genes, including those involved in the ROS scavenging system (Figure 5B) [122]. Recently, a total of 26 lncRNAs in salt-tolerant M-81E and 133 differentially expressed lncRNAs in salt-sensitive Roma strains of sweet sorghum were identified under salt-stress [123]. Strand-specific RNA-sequencing of salt-stressed root tissues of two closely related poplars (*Populus euphratica* and *Populus alba* var*. pyramidalis*) revealed a total of 10,646 and 10,531 lncRNAs in each [124]. Taken together, the identification of salt-responsive lncRNAs provides an opportunity to explore and improve our understanding of lncRNA-mediated gene regulation.

### 5.3. lncRNAs Controlling Nutrient Stress

The availability of certain nutrients in the soil can have severe effects on plant growth and development. Nutrient acquisition from the soil is one of the most critical physiological processes and mechanisms, including various membrane transporters involved in nutrient homeostasis [125]. Phosphorus (P) is one of the essential macronutrients contributing to plant growth and development, acting as a growth-limiting factor, and as a source of inorganic P (Pi) for ATP production [126]. However, the complex regulatory machines underlying P homeostasis in plants is scarce. Furthermore, plants have evolved several molecular and physiological mechanisms to improve Pi availability and P usage efficiency [127]. The role of lncRNAs in controlling phosphate availability in plants has been studied in several model plants such as *Arabidopsis* and rice [128,129]. In *Arabidopsis*, strand-specific RNA sequencing analysis of Pi-sufficient (P^+^) and Pi-deficient (P^−^) plants facilitated the identification of ~ 1212 lncRNAs, of which ~ 309 lncRNAs were differentially expressed under the mentioned conditions [128]. Npc536 is another lncRNA in *Arabidopsis* induced in leaves and roots by phosphate starvation and drought, which also affects root growth under salt stress conditions [112].

*PHOSPHATE2* (*PHO2*) is critical for Pi acquisition, translocation, and homeostasis in *Arabidopsis,* and miR399 mimics its expression [130]. *PHO2* expression is indispensable for Pi homeostasis when the Pi supply is ample. Upon Pi starvation, *PHO2* is suppressed by an activated level of miR399, which directs the cleavage of *PHO2* and thereby facilitates Pi uptake and translocation from root to shoot (Figure 5C). In *Arabidopsis*, the lncRNA *IPS*, sharing 23 nt complementary binding sites with miR399, acts as a target mimic for miR399 and controls the accumulation of target gene *PHO2* under Pi starvation, facilitating its uptake [45]. In this context, lncRNA *IPS* binds to miR399, preventing it from acting on its target gene *PHO2* when Pi is abundant in the soil. Elevated levels of *PHO2* abolish the functions of phosphate transporter 1 (*PHR1*) [25], thereby preventing Pi toxicity in roots by maintaining an optimum uptake of Pi. Under P^−^ conditions, miR399 directs the cleavage of the *PHO2* transcript, which presumably activates PHR’s function and eventually allows for Pi accumulation in the root. In *M. truncatula,* three lncRNAs—known as phosphate deficiency induced lncRNAs *(PDILs)*—have been identified to regulate Pi-deficiency signaling. Among them, *PDIL1* suppresses the degradation of *MtPHO2* by miR399 to regulate Pi deficiency signaling and Pi transportation, whereas *PDIL2*/*3* is involved in transcriptional regulation of Pi homeostasis [131].

Nitrogen (N) is another key essential nutrient for plant growth, development, and reproductive success. N serves as a major component of amino acids, energy-carrying molecule ATP (adenosine triphosphate), and N metabolism [132]. It is well established that N deficiency severely affects plant productivity, but molecular and genetic approaches have identified several regulatory and transporter genes controlling N use efficiency to help to improve plant productivity [133]. However, the molecular mechanism of N assimilation is not entirely known. Advancements in RNA-sequencing have further expanded our understanding of N-responsive lncRNAs’ roles in N homeostasis in plants. Studies showed that changes in N supply altered the expression of lncRNAs in rice [38], *Populus* [133], barley [134], and maize [41]. A study on lncRNAs in the rice genome under nitrogen starvation revealed the role of two cis-NAT-lncRNAs, *cis-NAT_AMT1.1_* and *cis-NAT_AMT1.2_,* derived from the sense strand of *AMT1* [38]. The regulatory mechanism of the nitrogen (N)-responsive lincRNA trans-acting siRNA3 *(TAS3)*, targeting the auxin response factor 4 (*ARF4)* gene, is well understood in *Arabidopsis* [87], whereas its possible new target—nitrate transporter (*NRT*)—needs further validation [135]. Similarly, a nitrate-induced lncRNA, *T5120*, is regulated by *NLP7,* a master regulator of nitrate signaling. *NLP*7 directly binds to nitrate-responsive cis-element (NRE)-like the motif in *T5120* and activates its transcription. Moreover, the expression of nitrate responsive gene *NRT1.1* was also upregulated. These findings suggest the NLP7-T5120-NRT regulatory module’s role in nitrate signaling and subsequently in improved nitrate assimilation and nitrogen use efficiency (Figure 5D) [136].

Among various micronutrients, Boron (B) is an essential micronutrient required for growth and development, in processes such as phenolic metabolism, nitrate assimilation, membrane integrity, and cell wall biosynthesis. The emerging evidence suggests that B’s molecular regulation is complicated, limited to coding genes, and attributed to lncRNAs, a novel regulatory player in the regulation of gene expression. A genome-scale exploration of lncRNAs in response to B toxicity detected ~8000 lncRNAs [137], whereas under B deficiency conditions in *P. trifoliata* 2101 lncRNAs were detected, of which 729 were upregulated and 721 were downregulated [138].

### 5.4. lncRNAs as Candidate Player in Cold Stress Tolerance

Temperature, either low or high temperature, is also essential to plant growth and productivity as a limiting factor. High-throughput RNA-sequencing has enabled researchers to elucidate several quantitative trait locus (QTLs) and various regulatory genes involved in cold tolerance in plants [139]. Likewise, several lncRNAs have emerged as potential genes involved in cold tolerance, and this has been well documented in various plant species. For instance, in *Arabidopsis*, weather-dependent reproductive-process-related lncRNAs *COOLAIR*, *COLDWRAP*, and *COLDAIR* facilitate the induction of flowering in the appropriate season [46,80,93].

Vernalization is a well-characterized phenomenon in flowering plants, causing them to flower in the favorable conditions of spring [140]. In *Arabidopsis*, *FLC* is a repressor of flowering. *COOLAIR*, a NAT-lncRNA transcribed from the sense transcript of *FLC*, controls *FLC* repression during vernalization [141]. *COLDAIR*, transcribed from the first intron of the *FLC* gene, represses FLC locus’s expression through PRC2-mediated chromatin modification [46]. The *FLC*-promoter-derived lncRNA *COLDWRAP* could further interact with stable repression of the *FLC* during vernalization [140]. The operating mechanisms of cold-induced *SVALKA* and cryptic antisense *CBF1* (*asCBF1*) lncRNAs have further advanced our understanding of the cold acclimation mechanism in plants [142]. In response to cold, *MAS* activated transcription of the corresponding cold responsive *MAF4,* recruiting the WDR5a complex for epigenetic modification at the *MAF4* locus for its activation and subsequently suppressed flowering [95].

### 5.5. lncRNAs as Emerging Regulators in Plant Responses to Biotic Stresses

Plants have evolved a multi-layered defense system to counteract pathogens through inducible or constitutive defenses. These defense responses include physical/chemical barriers, pattern recognition receptors (PRRs), and nucleotide-binding site–leucine-rich repeats (NBS-LRRs). In contrast, immunity-related lncRNAs are less documented in the plants. However, recent advanced sequencing technology has disclosed the role of lncRNAs in plant–pathogen interactions. For instance, in tomatoes, NBS-LRRs are a major regulatory component of the response to pathogen invasion. miR482b regulates NBS-LRR genes during infection. lncRNA23468 prevents the pairing of miR482b and encourages the accumulation of NBS-LRRs in tomato plants infected with *P. infestans* [143]. A very recent example is *ELENA1* lncRNA, which enhances resistance against disease caused by *P. syringae* and is involved in imparting plant immunity [24]. Similarly, lncRNA S-slylnc0957 acts as a negative regulator of TYLCV infection [144]. In wheat, 125 lncRNAs were responsive to powdery mildew infection [145]. Cui et al. [146] demonstrated that tomato *lncRNA16397* is an antisense lncRNA transcript of *SlGRX* that enhances resistance to *P. infestans* by reducing reactive oxygen species (ROS) accumulation. Many other biotic stress-related lncRNAs have been identified, but their biological roles are yet to be investigated.

miR482 acts as a negative regulator of NBS-LRR genes [147]. Cui et al. [148] reported that 88 lncRNAs predicted to decoy 46 miRNAs could target 30 coding genes, particularly NBS-LRR genes [147]. Among these lncRNA-miRNA-mRNA regulatory modules, *Sl-lncRNA15492-*miR482-NBS-LRR affects innate immunity by maintaining Sl-NBS-LRR homeostasis. [149]. The *Sl-lncRNA15492* lncRNA is 511 bp long, and pre-Sl-miR482a was located on the antisense sequence. *Sl-lncRNA15492* acts as a positive regulator and Sl-miR482a acts as a negative regulator of tomatoes’ resistance to *P. infestans.* In this context, *Sl-lncRNA15492* suppresses the expression of pre-Sl-miR482a to relieve the suppression of NBS-LRR genes and enhance tomato resistance against infection. However, when optimum levels of Sl-NBS-LRR accumulate, Sl-miR482a cleaves *Sl-lncRNA15492*, leading to an abundance of mature Sl-miR482a and decreasing the accumulation of NBS-LRR (Figure 5E) [149].

Considering the mounting evidence of lncRNAs in various biotic and abiotic stress responses in several plant species, researchers have provided novel insights into stress-responsive lncRNAs (SR-lncRNAs). However, the mechanisms involved in controlling the regulation of SR-lncRNAs are largely unknown and need further research. Table 3 presents several identified lncRNAs, along with their possible targets and mechanism of action contributing to the biotic and abiotic stress response in plants (Table 3).

## 6. Conclusions and Future Prospective

lncRNAs are among the most important types of ncRNAs emerging as potentially important players in various fundamental biological processes in both animals and plants. The development of high-throughput screening and other new techniques has helped to tailor the identification of lncRNAs and their biology. By contrast, our understanding of the regulation of synthesis and biogenesis of lncRNAs in plants remains scant. To date, the roles of very few plant lncRNAs have been explored. It has been found that lncRNAs are involved in induced responses to various abiotic and biotic stresses and that they play a crucial role in regulating gene expression during stress responses. The fine regulation of lncRNAs is essential for functional stress responses, and various studies have reported that the regulation of lncRNAs is associated with an array of responses. Emerging evidence suggests their regulatory roles in morphogenesis, tolerance to stress, response to pathogenic infection, and so on. As research into lncRNAs moves forward, aiming to uncover the regulatory mechanisms by which these lncRNAs act, novel functions in addition to those described here will likely be uncovered.

RNA-binding protein interactions play a pivotal role throughout the life of plants and are crucial for maintaining cellular processes and pathogenesis. LncRNA interactions with proteins may prove useful, and identifing proteins associated with lncRNA-mediated biological processes is critical. Consequently, the extension of sophisticated approaches like RNA-binding would undoubtedly improve our understanding of lncRNA biology and their systematic characterization. Loss and gain of function, RNA interference (RNAi), virus-induced gene silencing (VIGS), and recently developed systems such as transcription activator-like effector nucleases (TALENs) and clustered regulatory interspaced short palindromic repeats (CRISPR)/Cas9 systems can be effectivity employed to determine the precise function of lncRNAs in plants. Therefore, we suggest that lncRNAs may be developed into valuable molecular biology tools in plants to effectively control their response to various stresses. Increasing our understanding of precisely how lncRNAs regulate growth and development and stress responses will help to identify novel targets that will pave a path for developing new strategies for many plant processes.

## Figures and Tables

**Table 1:** List of genome-scale identifications of plant-specific lncRNAs in various species and under different abiotic and biotic conditions. EST: expressed sequence tag.

Name of Plants	No. of lncRNAs	Identification Approach	Stress
*Actinidia chinenesis*	7051	Transcriptome data	
*Arabidopsis*	1212	Transcriptome data	Phosphate starvation, drought stress, salt stress Heat, cold, salt,
	76	Genome data	
*B. napus*	3181	Transcriptome data	Cold and heat
	10,001	Transcriptome data	Sclerotinia stem rot by *Sclerotinia sclerotiorum*
*C. arietinum*	2248	Transcriptome data	
*C.s sativus*	3274	Transcriptome data	
*D. purpurea*	2660	Transcriptome data	Cold, dehydration
*F. vesca*	5884	Transcriptome data	
*G. arboretum*	5996	Transcriptome data	
*G. hirsutum*	1117	Transcriptome data	Salt
*H. annus*	25,327	Transcriptome data	
*M. truncatula*	503	EST and Genome data	Osmotic and salt
	23,324	Transcriptome data	
*M. notabilis*	1133	Transcriptome data	
*M. acuminata*	905	Transcriptome data	Drought, *Fusarium* wilt disease
	5294	Transcriptome data	
*O sativa*	2224	Transcriptome data	
*P. ginseng*	3688	Transcriptome data	
*P. trichocarpa*	2542	Transcriptome data	Drought
*Populus* sp.	7655	Transcriptome data	
*P. tomentosa*	1377	Transcriptome data	
*P. persica*	1417	Deep Sequencing	
*S. miltiorrhiza*	5446	Transcriptome data	Methyl-jasmonate
*S. italica*	584	Transcriptome data	Drought
*S. lycopersicum*	3679	Transcriptome data	
	1565	Transcriptome data	
*S. tuberosum*	1113	Transcriptome data	
*T. aestivum*	58,218	Transcriptome data	Heat stress, powdery mildew
	125	Transcriptome data	
*Z. mays*	1223	Transcriptome data	Drought stress
	20,163	Genome and transcriptome data	
	664	Genome data	
	7245	Transcriptome data	

**Table 2:** A summarized list of bioinformatics tools, resources, and archived databases predicting lncRNAs in plants.

	Name	Description	Weblink
**Prediction tools**	PhlyoCSF [53]	lncRNA coding potential calculator using codon substitution frequency (CSF) scores	https://github.com/mlin/PhyloCSF
	CPC [52]	lncRNA coding potential using sequence features and support vector machine (SVM)	http://cpc.cbi.pku.edu.cn/
	CNCI [61]	Calculation of lncRNA coding potential by profiling adjoining nucleotide triplets	https://github.com/www-bioinfo-org/CNCI
	CPAT [54]	Calculation of lncRNA coding potential using a logistic regression model	http://rna-cpat.sourceforge.net/
	DeepLNC [56]	lncRNA prediction using deep neural network	http://bioserver.iiita.ac.in/deeplnc/
	iSeeRNA [55]	lncRNA prediction using SVM algorithm	http://137.189.133.71/iSeeRNA/
	lncRNATargets [57]	lncRNA target prediction based on nucleic acid thermodynamics	http://www.herbbol.org:8001/lrt/index.php
	spongeScan [58]	detection of miRNA elements in sponge lncRNAs	http://spongescan.rc.ufl.edu/
	TF2LncRNA [60]	Identification of transcription factors for lncRNAs	http://mlg.hit.edu.cn/tf2lncrna
	RegRNA [59]	For identifying regulatory RNA motifs	http://regrna2.mbc.nctu.edu.tw/index.html
**Database**	NONCODE [62]	Complete collection and annotation of ncRNAs from 16 species, including *Arabidopsis* as the only plant species; 3853 lncRNA transcripts and 2477 lncRNA genes	http://www.noncode.org/
	GREENC [63]	A wiki-based plant lncRNA database from 37 plant species; 120000 lncRNAs	http://greenc.sciencedesigners.com/
	lncRNAdb [64]	Literature describing functions of lncRNAs from *Arabidopsis*, rice, and others	http://lncrnadb.org
	PLncDB [32]	*Arabidopsis* lncRNAs; >13000 lncRNAs	http://chualab.rockefeller.edu/gbrowse2/homepage.html
	PNRD [65]	lncRNAs from *Arabidopsis*, poplar, maize, and rice; 5573 lncRNAs	http://structuralbiology.cau.edu.cn/PNRD/
	CANTATAdb [66]	lncRNAs from 10 model plant species; 45117 lncRNAs	http://yeti.amu.edu.pl/CANTATA/
	PLNlncRbase [67]	Experimentally verified plant lncRNAs in 43 species: 1187 lncRNAs	http://bioinformatics.ahau.edu.cn/PLNlncRbase/pcsb
	PLncRNAdb [68]	Plant-specific lncRNAs with a distinct annotation like information lncRNAs and various RNA-binding proteins (RBPs); 5000 lncRNAs	http://bis.zju.edu.cn/PlncRNADB/index.php
	EVLncRNAs [69]	Experimentally validated lncRNAs in 77 plants and animals; 1543 lncRNAs	http://biophy.dzu.edu.cn/EVLncRNAs

**Table 3:** Representative studies showing lncRNA-mediated growth, development, and stress tolerance in plants over the last decade.

Role	Species	lncRNA	Function	Reference
**Development**	*M. truncatula*	*ENOD40*	Nodule development	[14]
	*Arabidopsis*	*COOLAIR*	Flowering	[21]
	*B. campestris*	*MF11*	Male sterility	[22]
	*B. campestris*	*ZM40*	Male sterility	[23]
	*Arabidopsis*	*APOLO*	Polar auxin transport	[26]
	*Arabidopsis*	NATs	Photomorphogenesis	[33]
	*Arabidopsis*	*COLDAIR*	Flowering	[46]
	*O. sativa*	*LDMAR*	Male sterility	[47]
	*Arabidopsis*	*HIDI*	Photomorphogenesis	[69]
	*Arabidopsis*	*COLDWRAP*	Flowering	[80]
	*Arabidopsis*	*ASCO*	Regulation of auxin	[82]
	*Arabidopsis*	*MAS*	Flowering	[95]
	*Arabidopsis*	*CDF5*	Circadian	[96]
	*Arabidopsis*	*AtR8*	Hypoxic stress	[96]
	*Arabidopsis*	*FLORE*	Flowering	[96]
	*Arabidopsis*	*LINC*-*AP2*	Flowering	[98]
	*M. polymorpha*	*SUF*	Sexual differentiation	[100]
	*O. sativa*	*MIKKI*	Root Growth	[107]
	*S. buckthorn*	*LNC1*/2	Anthocyanin biosynthesis	[150]
	*Arabidopsis*	*asHSFB2a*	Vegetative and gametophytic development	[151]
	*C. sativus*	*CsM10*	Sex differentiation	[152]
	*G. max*	*GmENOD40*	Nodule development	[153]
	*S. lycopersicum*	*lncRNA000170*	Trichome formation	[154]
	*S. lycopersicum*	*lncRNA1459*	Fruit ripening	[155]
	*H. vulgare*	*HvCesA6*	Cell wall synthesis	[156]
	*P. hybrid*	*SHO*	Cytokinin biosynthesis	[157]
	*Arabidopsis*	*ASL*	Flowering	[158]
**Biotic and abiotic stress**	*Arabidopsis*	*ELENA1*	Pathogen infection	[24]
	*Arabidopsis*	*IPS1*	Phosphate starvation	[45]
	*Arabidopsis*	*T5120*	Nitrate assimilation	[136]
	*Arabidopsis*	*SVALKA*	Cold-induced	[142]
	*S. lycoperscium*	*lncRNA23468, lncRNA16397*	*P. infestans* infection	[143,146]
	*S. lycoperscium*	*S-slylnc0957*	Pathogen infection	[144]
	*Arabidopsis*	*DRIR*	Drought tolerance	[159]
	*M. truncatula*	*Mt4*	Phosphate starvation	[160]
	*O. sativa*	*OsPI1*	Phosphate starvation	[161]
	*O. sativa*	*ALEX1*	JA-mediated disease resistance	[162]
	*S. lycoperscium*	*TPS11*	Phosphate starvation	[163]
	*Z. mays*	*PILNCR1*	Phosphate starvation	[164]
	*N. attenuata*	*JAL1/3*	JA-mediated herbivore resistance	[165]

## Data Availability

No new data or existing data were created/used or analyzed in this study.

## Abbreviations

lncRNAs	Long non-coding RNAs
sRNAs	Small RNAs
miRNAs	MicroRNAs
ENOD4	Early nodulin 40
Igf2	insulin-like growth factor 2
TYLCV	Tomato yellow leaf curl virus
SAGE	Serial expression of gene expression
EST	Expressed sequence tag
NATs	Natural antisense transcripts
TTS	Transcriptional termination site
TCV	Turnip crinkle virus
SCL	Scarecrow like
NF-Y	Nuclear factor Y
N	Nitrogen
B	Boron
P	Phosphorous
Pi	inorganic Phosphorous
